# Bioinformatics analysis of the potential biomarkers for acute respiratory distress syndrome

**DOI:** 10.1042/BSR20192436

**Published:** 2020-09-04

**Authors:** Lin Liao, Pinhu Liao

**Affiliations:** School of Medicine, Guangxi University, Nanning, Guangxi Zhuang Autonomous Region, China

**Keywords:** ARDS, Biomarker, Prognosis, SPP1

## Abstract

Background: Acute respiratory distress syndrome (ARDS) is caused by uncontrolled inflammation, and the activation of alveolar macrophages (AM) is involved in pathophysiologic procedures. The present study aimed to identify key AM genes and pathways and try to provide potential targets for prognosis and early intervention in ARDS. Methods: The mRNA expression profile of GSE89953 was obtained from the Gene Expression Omnibus database. The LIMMA package in R software was used to identify differentially expressed genes (DEGs), and the clusterProfiler package was used for functional enrichment and pathway analyses. A protein–protein interaction network of DEGs was constructed to identify hub genes via the STRING database and Cytoscape software. Hub gene expression was validated using differentially expressed proteins (DEPs) obtained from the ProteomeXchange datasets to screen potential biomarkers. Results: A total of 166 DEGs (101 up-regulated and 65 down-regulated) were identified. The up-regulated DEGs were mainly enriched in regulation of the ERK1 and ERK2 cascade, response to interferon-gamma, cell chemotaxis, and migration in biological processes. In the KEGG pathway analysis, up-regulated DEGs were mainly involved in rheumatoid arthritis, cytokine–cytokine receptor interactions, phagosome, and the chemokine signaling pathway. The 12 hub genes identified included GZMA, MPO, PRF1, CXCL8, ELANE, GZMB, SELL, APOE, SPP1, JUN, CD247, and CCL2. Conclusion: SPP1 was consistently differentially expressed in both DEGs and DEPs. SPP1 could be a potential biomarker for ARDS.

## Introduction

Acute respiratory distress syndrome (ARDS) is an acute inflammatory lung injury characterized by a rapidly progressing respiratory failure, pulmonary edema, diffuse alveolar damage, and inflammatory cell infiltration [1]. Mortality of ARDS has decreased due to the development of treatment methods, such as the protective mechanical ventilation strategy, but remains high. Prognosis evaluation and early intervention in ARDS patients at high risk would benefit the clinic outcome. Previous studies suggested that multiple plasma biomarkers, such as IL-6, IL-8, angiopoietin-2 (ANG-2), Kerbs von Lungren 6 antigen (KL-6), receptor for advanced glycation endproducts (RAGEs), and surfactant protein D (SP-D), had a predictive value for ARDS prognosis [1–4]. Nevertheless, bronchoalveolar lavage fluid (BALF) is likely to provide more timely and detailed biomarkers for evaluating the degree of lung injury. The ARDS prognostic biomarkers identified in BALF may be more valuable than those in blood.

Alveolar macrophages (AM) are one of the most important regulatory cells in lung inflammation [5]. Previous studies indicated that AM had a critical impact on occurrence and development of ARDS [6–8]. Therefore, the expressions of AM-related genes and proteins have the potential to be used as therapeutic targets and prognostic biomarkers in ARDS.

In the present study, differentially expressed genes (DEGs) of AM from ARDS patients were analyzed to evaluate the potential targets for prognosis and early intervention for ARDS.

## Materials and methods

### Overview of data

The mRNA expression profile of GSE89953 was downloaded from the GEO (http://www.ncbi.nlm.nih.gov/geo) database. The GSE89953 dataset was submitted by Morrell et al. and included 26 paired AM and peripheral blood monocytes (MONO) samples from ARDS patients [9]. The proteomic profile (PXD002672) in the present study was submitted by Bhargava et al. [10] and was deposited in the ProteomeXchange (http://proteomecentral.proteomexchange.org). BALF samples were obtained from 20 surviving and 16 non-surviving ARDS patients, and iTRAQ was used to analyze the protein expression profiles of these samples.

### Differentially expressed genes identifying

The LIMMA package (version 3.38.3) in R software was used to identify DEGs between paired AM and MONO samples in 26 ARDS patients. Adjusted *P* value <0.05 and | logFC| >2 were set as the threshold. Biclustering analysis for the DEGs was performed using pheatmap package in R software.

### Functional enrichment and pathway analysis

The clusterProfiler package (version3.5) in R software was used for Gene Ontology (GO) and Kyoto Encyclopedia of Genes and Genomes (KEGG) pathway enrichment analysis of DEGs [11]. *P* values < 0.05 showed significant enrichment.

### Protein–protein interaction network construction and hub genes establishment

The DEGs were submitted to the online Search Tool for Retrieval of Interacting Genes/Proteins database (STRING, Version 11.0, http://www.string-db.org/) for protein–protein interaction (PPI) analysis. All active interaction sources were retained, and a minimum required interaction score = 0.4 was considered significant. The interaction data were subsequently imported into the Cytoscape software (version 3.6.1) to visualize the PPI network. The hub genes were identified by finding the intersections of the top 20 genes from 12 algorithms using the Cytoscape plugin CytoHubba (version 0.1).

### Screening for prognostic biomarkers

The RAW files from proteomic profile were processed using ProteinPilot software (version 4.5), a maximum false discovery rate of 5% was required for all reported proteins. Proteins with |logFC|>0.2 and *P* value <0.05 were considered as differentially expressed proteins (DEPs) between survivors and non-survivors groups. The higher proteins in the survivors indicated positive value. Overlap analysis of hub genes and proteomic data was done to screen potential AM biomarkers that could be used as prognostic markers for ARDS.

## Results

### Differential expression analysis

**Figure 1:**
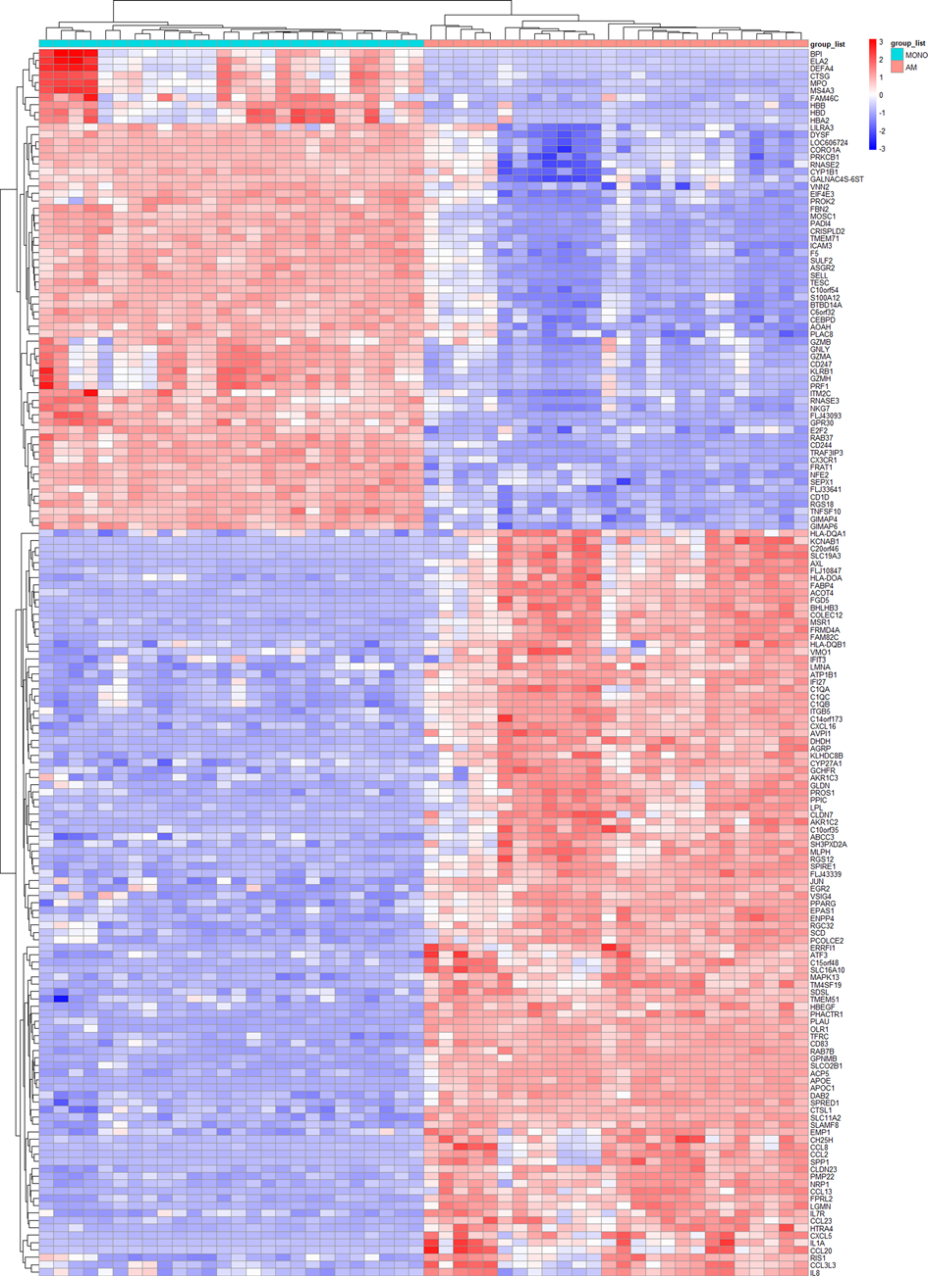
Heatmap plot of the 166 DEGs between alveolar macrophages and peripheral blood monocytes in GSE89953 The red indicates the up-regulated genes, the blue indicates the down-regulated genes.

A total of 166 DEGs were identified in AM and MONO in which 101 up-regulated and 65 down-regulated. The DEG expression heatmap is presented in Figure 1.

### Gene Ontology analysis and pathway enrichment analysis of DEGs

**Figure 2:**
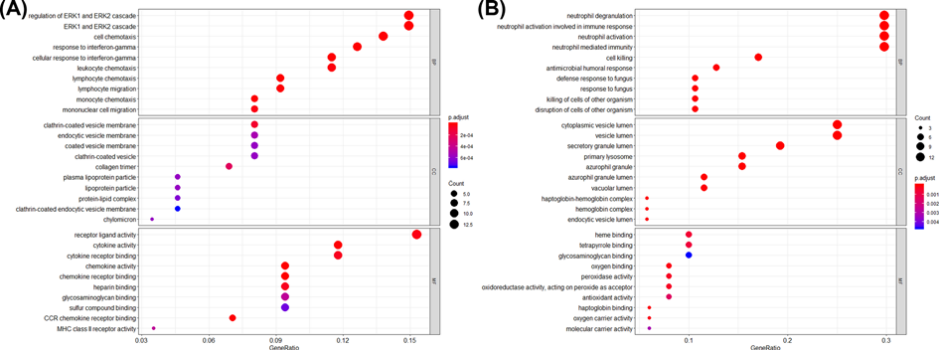
Functional enrichment analyses of the DEGs (**A**) GO enrichment analysis of the up-regulated DEGs. (**B**) GO enrichment analysis of the down-regulated DEGs; BP, biological process; CC, cellular component; MF, molecular function.

GO analysis indicated that the up-regulated DEGs were mainly involved in regulation of the ERK1 and ERK2 cascade, response to interferon-gamma, cell chemotaxis, and migration in the biological process (BP) (Figure 2A), and the down-regulated DEGs were primarily enriched in cell killing, neutrophil activation, degranulation, and mediated immunity (Figure 2B).

**Figure 3:**
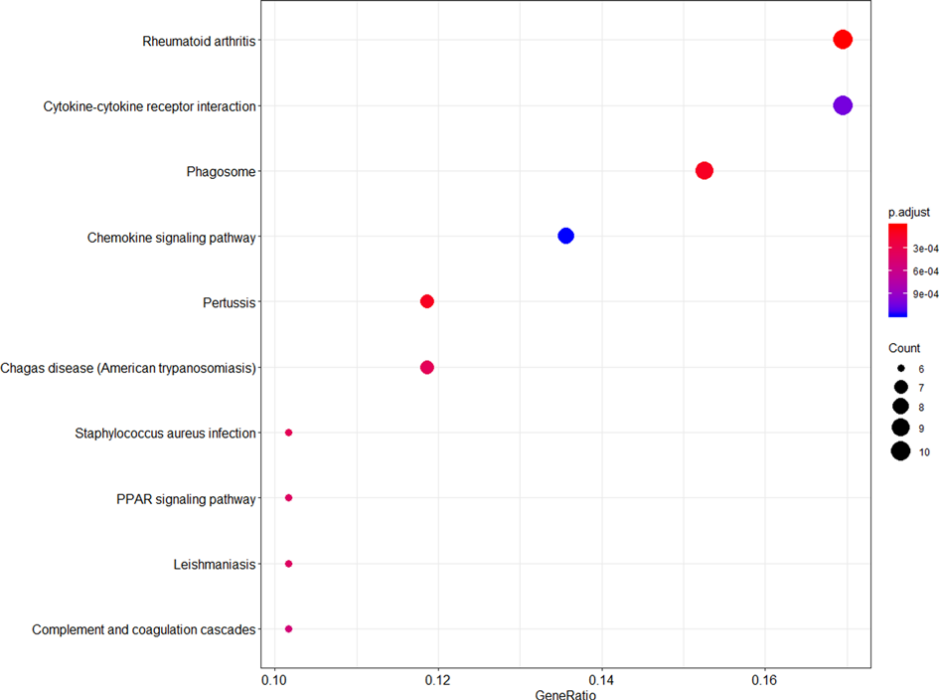
KEGG pathway enrichment analysis of the up-regulated DEGs The gradual color indicates the -log10 (*P*). The size of the dots indicates the gene count.

The KEGG pathway analysis indicated that the up-regulated DEGs were mainly involved in rheumatoid arthritis, cytokine–cytokine receptor interaction, phagosome, and chemokine signaling pathway (Figure 3 andTable 1), and the down-regulated DEGs were mainly involved in natural killer cell mediated cytotoxicity (Table 1).

**Table 1:** Pathway enrichment analysis of DEGs

Expression	Pathway ID	Description	*P*-value	Count
Up-regulated	hsa05323	Rheumatoid arthritis	1.04 × 10^−09^	10
	hsa05133	Pertussis	1.37 × 10^−06^	7
	hsa04145	Phagosome	1.66 × 10^−06^	9
	hsa05150	Staphylococcus aureus infection	1.06 × 10^−05^	6
	hsa05142	Chagas disease (American trypanosomiasis)	1.06 × 10^−05^	7
	hsa03320	PPAR signaling pathway	1.73 × 10^−05^	6
	hsa05140	Leishmaniasis	1.73 × 10^−05^	6
	hsa04610	Complement and coagulation cascades	2.52 × 10^−05^	6
	hsa04060	Cytokine–cytokine receptor interaction	5.73 × 10^−05^	10
	hsa04062	Chemokine signaling pathway	7.82 × 10^−05^	8
Down-regulated	hsa04650	Natural killer cell mediated cytotoxicity	4.87 × 10^−05^	5
	hsa05144	Malaria	4.77 × 10^−04^	3

### Construction of protein–protein interaction network and identification of hub genes

**Figure 4:**
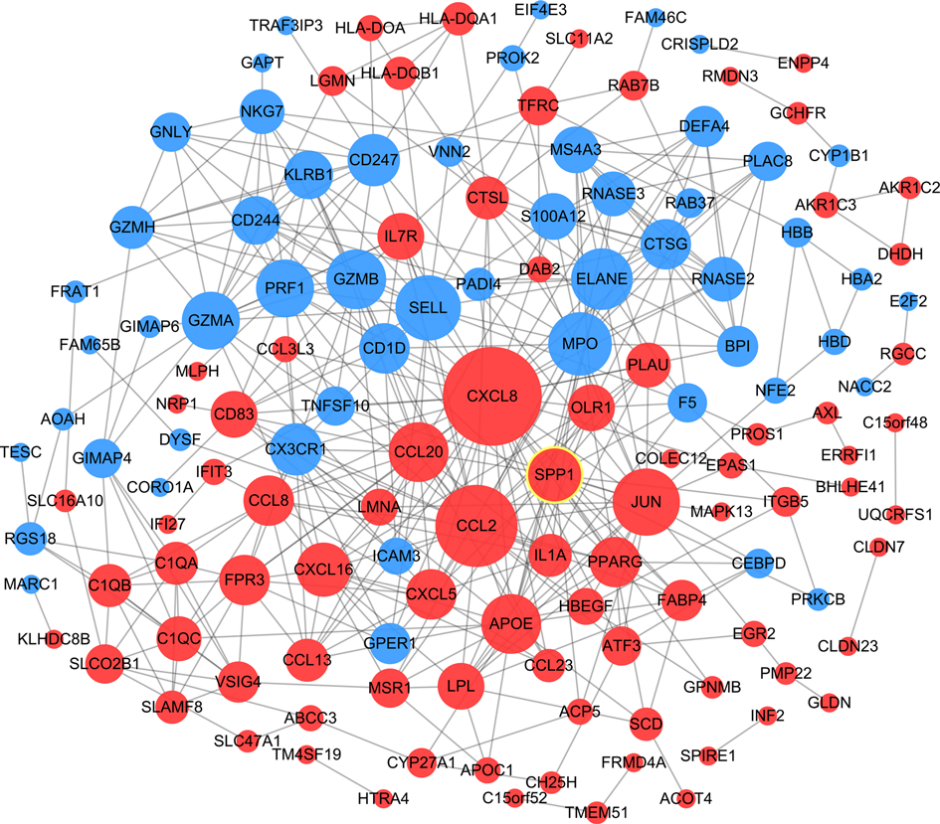
PPI network constructed with DEGs The red nodes indicate up-regulated genes, and the blue nodes indicate down-regulated genes. The higher the degree, the larger the node.

The PPI network was constructed by importing DEGs into the STRING database and Cytoscape software, which contained 163 nodes and 435 edges (Figure 4). The top 12 hub genes were identified using CytoHubba and included GZMA, MPO, PRF1, CXCL8, ELANE, GZMB, SELL, APOE, SPP1, JUN, CD247, and CCL2. CXCL8 showed the highest degree of 39.

### Prognostic biomarker validation

**Figure 5:**
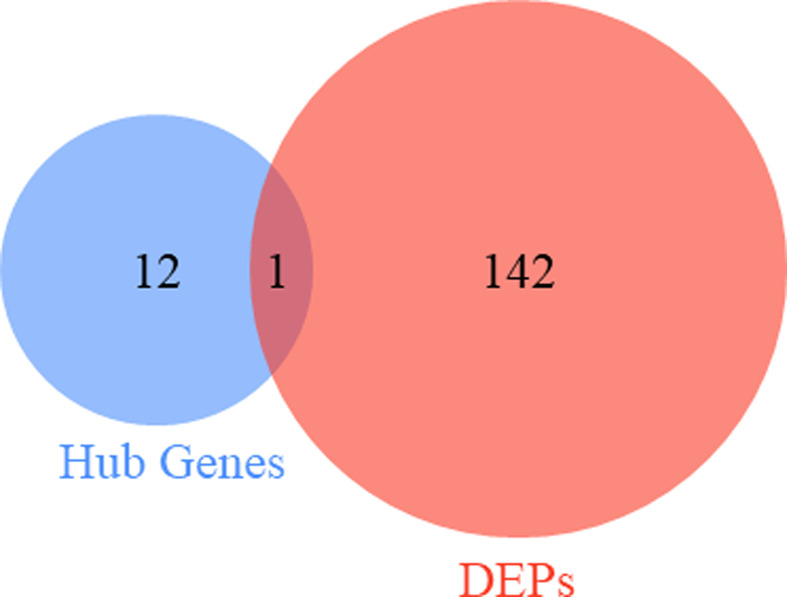
Venn diagram analysis of DEPs and hub genes The blue circle represents the hub genes, the red circle represents the DEPs. The intersection of the two circles represents potential prognostic biomarker.

By integrated analysis, a total of 142 DEPs from ARDS survivors and non-survivors were extracted from proteomic profile PXD002672. Potential prognostic biomarkers were identified by synthesizing the top 12 hub genes and the 142 DEPs via Venn diagram analysis. The results indicated that SPP1 was consistently differentially expressed in both datasets (Figure 5). SPP1 was different at the transcriptional and protein expression level.

## Discussion

At present, single omics technology was widely used in ARDS research, but it was not enough to elucidate the complicated pathophysiological mechanism of ARDS. Therefore, multi-omics integrated analysis has provided more comprehensive and systematic understanding of ARDS occurrence and development, which is conducive to the discovery of new biomarkers and therapeutic targets.

ARDS is an life-threatening lung injury common in intensive care units. Despite the rapid development of diagnostic and treatment technology, the high morbidity and mortality of ARDS have not significantly improved. Identifying potential biomarkers for early intervention and prognosis of ARDS is important to improve clinic outcomes. In the present study, 166 DEGs were identified between paired AM and MONO samples in 26 ARDS patients, including 101 up-regulated genes and 65 down-regulated genes. Up-regulated genes were mainly involved in regulation of ERK1 and ERK2 cascade, response to interferon-gamma, cell chemotaxis, and migration. This is consistent with earlier studies showing that enhancement of AM immune and inflammatory responses plays a crucial role in ARDS development and progression [12–14]. The most significantly enriched of the KEGG pathway up-regulated DEGs were rheumatoid arthritis, cytokine–cytokine receptor interaction, phagosome, and the chemokine signaling pathway. Previous reported research have shown that these pathways are involved in AM phagocytosis, cytotoxicity, and chemotaxis [15,16].

The construction of the PPI network to identify 12 hub genes associated with ARDS suggested that CXCL8 (IL-8), APOE, SPP1, JUN, and CCL2 were significantly up-regulated, and GZMA, MPO, PRF1, ELANE, GZMB, SELL, and CD247 were significantly down-regulated. IL-8 and CCL2, as chemokines, are directly involved in the occurrence and development of ARDS [17]. IL-8 is considered to be the most important chemokine that recruits neutrophils to inflammatory sites [18], and CCL2 specifically recruits peripheral blood monocytes [19]. A previous study showed that GZMB deficiency increased morbidity and mortality in bleomycin-induced acute lung injury model in mice [20]. The results from Venn analysis revealed that the level of SPP1 expression in AM was higher in non-survive ARDS patients than those who did survive.

Osteopontin (OPN), encoded by SPP1, is a widely distributed extracellular matrix protein, which is involved in various biological processes, including tumor progression, bone metabolism, and inflammation. In the respiratory system, OPN is mainly expressed in AM, bronchial epithelial cells, endothelial cells, and fibroblasts. OPN is involved in regulating chemotaxis and cytokine expression, as well as inflammatory and immunological responses, promotes lung injury by regulating adhesion and migration of neutrophils [21,22]. It has been confirmed that the level of OPN was positively correlated with disease severity in COPD patients with emphysema, the OPN was located in AM by immunohistochemistry staining [23]. Other researchers showed that OPN is mainly expressed in proliferating alveolar epithelial cells in idiopathic pulmonary fibrosis, induces proliferation and migration of fibroblasts and epithelial cells, and regulates the expression of MMPs and TIMPs to promote fibrosis [24]. OPN is strongly expressed in AM and promoted the expression of MMP7, TNF-α, and IL-6 while impairing the epithelial sodium channel, thus increasing influenza virus-induced lung injury [25]. OPN expressed by AM reduced NO production by inhibiting inducible NO synthase on lipopolysaccharide-induced ARDS in mice [26].

However, in a different study, OPN was found to be beneficial [27]. Recent research suggested that OPN reduced histone-induced lung injury via binding to histones [28]. Although OPN exhibits both pro-inflammatory and anti-inflammatory effects, inflammatory mediators and OPN comprise a complex network, leading to uncontrolled inflammation in the lungs during the occurrence and development of ARDS. From a therapeutic perspective, neutralization of OPN could improves the outcome of ARDS. More specifically, treatment with an anti-OPN antibody protects against sepsis-induced acute lung injury in mice by inhibiting neutrophil migration in the lungs [22]. Injection of anti-major histocompatibility complex class I antibodies caused transfusion-related acute lung injury that was ameliorated in wild-type mice using an anti-OPN antibody and in OPN knockout mice [29]. In addition, specifically blocking OPN receptors (integrins/CD44) and inhibiting transcription factors that initiate OPN expression may also be a strategy for the treatment of ARDS in the future [30,31].

## Conclusions

In conclusion, the present study used bioinformatic analyses to identify key genes and pathways to facilitate further understanding of the role of AM in ARDS. Integrated analysis revealed that SPP1 could be used as a predictive biomarker for ARDS. However, this needs to be confirmed by further experimental studies.

## Abbreviations

ANG-2: angiopoietin-2
ARDS: acute respiratory distress syndrome
AM: alveolar macrophages
BP: biological process
CC: cellular component
DEG: differentially expressed gene
DEP: differentially expressed protein
KL-6: Kerbs von Lungren 6 antigen
MF: molecular function
OPN: osteopontin
RAGE: receptor for advanced glycation endproducts
SP-D: surfactant protein D
